# The novel desmopressin analogue [V^4^Q^5^]dDAVP inhibits angiogenesis, tumour growth and metastases in vasopressin type 2 receptor-expressing breast cancer models

**DOI:** 10.3892/ijo.2015.2952

**Published:** 2015-04-03

**Authors:** JUAN GARONA, MARINA PIFANO, ULISES D. ORLANDO, MARIA B. PASTRIAN, NANCY B. IANNUCCI, HUGO H. ORTEGA, ERNESTO J. PODESTA, DANIEL E. GOMEZ, GISELLE V. RIPOLL, DANIEL F. ALONSO

**Affiliations:** 1 Laboratory of Molecular Oncology, National University of Quilmes, Bernal, B1876BXD, Argentina; 2 Biomedical Research Institute (INBIOMED), Department of Human Biochemistry, School of Medicine, University of Buenos Aires, Buenos Aires, Argentina; 3 School of Pharmacy and Biochemistry, University of Buenos Aires, Buenos Aires, Argentina; 4 Institute of Veterinary Sciences (ICIVET-CONICET), National University of Litoral, Esperanza, Santa Fe, Argentina

**Keywords:** desmopressin analogue, [V^4^Q^5^]dDAVP, breast carcinoma, vasopressin type 2 receptor, metastases, tumour vascularisation

## Abstract

Desmopressin (dDAVP) is a safe haemostatic agent with previously reported antitumour activity. It acts as a selective agonist for the V2 vasopressin membrane receptor (V2r) present on tumour cells and microvasculature. The purpose of this study was to evaluate the novel peptide derivative [V^4^Q^5^]dDAVP in V2r-expressing preclinical mouse models of breast cancer. We assessed antitumour effects of [V^4^Q^5^]dDAVP using human MCF-7 and MDA-MB-231 breast carcinoma cells, as well as the highly metastatic mouse F3II cell line. Effect on *in vitro* cancer cell growth was evaluated by cell proliferation and clonogenic assays. Cell cycle distribution was analysed by flow cytometry. In order to study the effect of intravenously administered [V^4^Q^5^]dDAVP on tumour growth and angiogenesis, breast cancer xenografts were generated in athymic mice. F3II cells were injected into syngeneic mice to evaluate the effect of [V^4^Q^5^]dDAVP on spontaneous and experimental metastatic spread. *In vitro* cytostatic effects of [V^4^Q^5^]dDAVP against breast cancer cells were greater than those of dDAVP, and associated with V2r-activated signal transduction and partial cell cycle arrest. In MDA-MB-231 xenografts, [V^4^Q^5^]dDAVP (0.3 μg/kg, thrice a week) reduced tumour growth and angiogenesis. Treatment of F3II mammary tumour-bearing immunocompetent mice resulted in complete inhibition of metastatic progression. [V^4^Q^5^]dDAVP also displayed greater antimetastatic efficacy than dDAVP on experimental lung colonisation by F3II cells. The novel analogue was well tolerated in preliminary acute toxicology studies, at doses ≥300-fold above that required for anti-angiogenic/antimetastatic effects. Our data establish the preclinical activity of [V^4^Q^5^]dDAVP in aggressive breast cancer, providing the rationale for further clinical trials.

## Introduction

Desmopressin (1-deamino-8-D-arginine vasopressin or dDAVP) is a synthetic peptide derivative of the antidiuretic hormone used to boost the levels of clotting factors in certain haemostatic disorders (1). DDAVP differs from the natural peptide by deamination of cystein in position 1, which prolongs its half-life, and substitution of L-arginine by D-arginine in position 8, which reduces the pressor effect and confers selectivity for the vasopressin type 2 membrane receptor (V2r) (2). This receptor subtype is present in kidney collecting ducts and endothelium (3,4). By acting on endothelial cells dDAVP induces a strong haemostatic effect causing the release of coagulation factor VIII, von Willebrand factor (VWF) and plasminogen activators from microvascular stores into the bloodstream (5). V2r expression was also reported in transformed epithelial cells and several human tumour cell lines, including breast cancer (6,7). V2r stimulation in breast carcinoma is associated with antiproliferative signalling, involving activation of adenylate cyclase followed by intracellular cAMP elevation (8).

**Figure 1:**
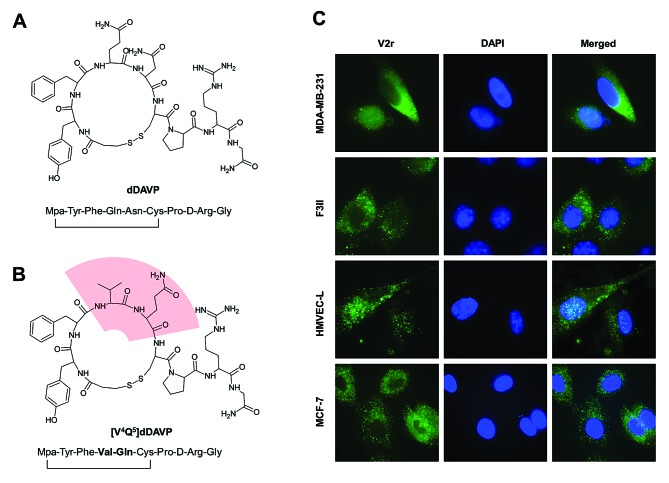
Peptide sequence of [V^4^Q^5^]dDAVP and immunofluorescence detection of vasopressin type 2 receptor in breast cancer and microvascular endothelial cell lines. Schematic view of (A) parental compound dDAVP (1-deamino-8-D-arginine vasopressin) and (B) novel analogue [V^4^Q^5^]dDAVP (1-deamino-4-valine-5-glutamine-8-D-arginine vasopressin). Red shaded area indicates site of amino acid substitution belonging to the loop region of the peptide. Amino acid sequences are shown using the standard three-letter designations. Disulfide bonds between positions 1 and 6 are shown with connecting lines. Bold type text indicates modified amino acids in positions 4 and 5. (C) Detection of V2r expression by immunofluorescence. MDA-MB-231 human breast carcinoma cells, HMVEC-L human microvascular endothelial cells from lung, F3II mouse mammary carcinoma cells and MCF-7 human breast carcinoma cells (positive control) are shown. Magnification, ×1,000.

Preclinical studies in mice showed that intravenous administration of dDAVP inhibited experimental lung metastases in a dose-dependent manner (9,10) and dramatically decreased locoregional and distant spread in a model of surgical manipulation of aggressive breast tumours (11). Hermo *et al* confirmed the beneficial effect of perioperative dDAVP on survival in dogs with advanced mammary cancer (12,13). As mentioned above, dDAVP drastically increases circulating levels of VWF by acting on V2r in endothelial cells. Terraube and collaborators showed that VWF plays a protective role against cancer cell dissemination and absence of VWF leads to increased metastatic potential (14). Additionally, our group reported that dDAVP inhibited the early angiogenic response and markedly decreased vascularisation of growing subcutaneous tumours (15). Experimental evidence suggested that dDAVP reduces angiogenesis by inducing the formation of angiostatin, a potent inhibitor of angiogenesis that is generated by cancer-mediated proteolysis of plasminogen (16,17). Thus, dDAVP seems to produce a dual antimetastatic and anti-angiogenic effect, breaking the cooperative interplay of tumour and endothelial cells during disease progression (18). Taken together, dDAVP appears as a promising lead compound for the development of novel peptide analogues with enhanced anticancer efficacy. With this purpose, dDAVP (Fig. 1A) was rationally modified, and the novel analogue [V^4^Q^5^]dDAVP (1-deamino-4-valine-5-glutamine-8-D-arginine vasopressin) (Fig. 1B) was synthesized and assayed. Amino acid positions 4 and 5 belong to the conformational peptide loop which has a key role in ligand-receptor interaction and antitumour activity (5,19–22). In an initial evaluation, [V^4^Q^5^]dDAVP exhibited a significantly higher cytostatic effect against breast cancer cells than the parental compound dDAVP and compared to other screened peptide derivatives (21). In the present study, we further characterized the anticancer activity of the novel analogue [V^4^Q^5^]dDAVP on V2r-expressing breast cancer preclinical models. The effect of the compound on xenograft tumour growth and angiogenesis was assessed. Additionally, we determined the efficacy of the novel analogue on metastatic progression in immunocompetent hosts.

## Materials and methods

### Cell lines and culture conditions

Human breast carcinoma cell lines MDA-MB-231 (ATCC HTB-26) and MCF-7 (ATCC HTB-22) were obtained from the American Type Culture Collection. MDA-MB-231 is a triple-negative breast cancer (TNBC) cell line which lacks the oestrogen receptor (ER) and progesterone receptor (PR), and expresses low levels of human epidermal growth factor receptor 2 (HER2)/neu. It also belongs to the claudin-low molecular subtype. MCF-7 is a ER-positive/PR-positive luminal mammary carcinoma (23). The F3II mammary carcinoma cell line is a highly invasive and metastatic variant derived from a clone of a spontaneous BALB/c mouse mammary tumour. It is a hormone-independent tumour cell line and express low levels of HER2/neu. Tumour cells were grown in Dulbecco’s modified Eagle’s medium (DMEM, Gibco, Rockville, MD, USA) plus 10% fetal bovine serum (FBS), 2 mM glutamine and 80 μg/ml gentamycin in monolayer culture, at 37°C in a humidified atmosphere of 5% CO_2_. HMVEC-L human microvascular endothelial cell line was obtained from Cascade Biologics and cultured in gelatin coated plates using endothelial cell medium with specific growth factors (EGM-2 MV Bullet Kit, Lonza, Milan, Italy). All cells were harvested using a tripsin/EDTA solution (Gibco) diluted in phosphate-buffered saline (PBS).

### Immunofluorescence detection of V2r

Briefly, cells were seeded on glass coverslips, and fixed with paraformaldehyde. After incubation with blocking agent, cells were incubated with a goat polyclonal anti-V2r antibody for 1 h at 37°C (Santa Cruz Biotechnology, Santa Cruz, CA, USA). Receptor-bound antibodies were detected with a secondary rabbit polyclonal FITC-conjugated antibody (Chemicon International, Temecula, CA, USA) and nuclei were labeled with DAPI (Vector Laboratories, Peterborough, UK). Samples were examined using a TE-2000 microscope (Nikon Inc., Tokyo, Japan). MCF-7 cells were used as a positive control of V2r expression (6).

### Peptide compounds and dosing

DDAVP and [V^4^Q^5^]dDAVP were synthesized in the solid phase, using Nα-Fmoc protection and the tea-bag strategy (21). Peptides were purified by reversed-phase high-performance liquid chromatography and quantified using a commercial dDAVP reference standard (BCN Peptides, Barcelona, Spain).

Compounds were injected intravenously at 0.3 μg/kg, this being a clinically relevant dose of dDAVP with widely acknowledged haemostatic effects in humans (4), as well as antitumour properties in mice (10). Since it is known that peptides such as dDAVP can induce tachyphylaxis with daily applications (3), compounds were administered on a thrice-weekly basis when treatment lasted for >5 days. *In vitro* experiments were performed using nanomolar and low micromolar concentrations of the peptides, a range consistent with the *in vivo* dosage (9,24).

### Cell proliferation assay

Antiproliferative effect against rapidly growing tumour cells was measured using the 3-(4,5-dimethylthiazol-2-yl)-2,5-diphenyltetrazolium bromide (MTT) assay (Sigma-Aldrich, St. Louis, MO, USA). Briefly, cells were plated in 96-well flat bottom plates at a density of 2.5×10^3^ per 200 μl in complete DMEM, allowed to attach overnight, and then treated with dDAVP or [V^4^Q^5^]dDAVP (100–1,500 nM) or vehicle for 72 h. Blockade of agonistic effect was achieved by incubation with the selective and competitive V2r antagonist tolvaptan (Otsuka Pharmaceutical Co., Tokyo, Japan). MTT reagent was added to each well and the plate incubated for 4 h. After solubilisation using dimethyl sulfoxide the absorbance of each well was measured at 570 nm. The optical density of untreated control cells was taken as 100% viability.

### cAMP quantification

Briefly, carcinoma cells were stimulated with [V^4^Q^5^]dDAVP (1,000 nM) or saline vehicle for 1 h and intracellular cAMP levels were measured using the Cyclic AMP EIA kit (ACE, Cayman Chemical Co., Ann Arbor, MI, USA) according to the manufacturer’s instructions.

### PKA activity

The effect of [V^4^Q^5^]dDAVP on PKA activity of MCF-7 total cell extract was determined by measuring the incorporation of [^32^P] orthophosphate from [^32^P]γ-ATP into PKA substrate histone H1. We tested the activity of the enzyme present in cells incubated for 30 min or 1 h with or without [V^4^Q^5^]dDAVP (1,000 nM) or 8-Br-cAMP (500 μM). 8-Br-cAMP is a membrane-permeable analogue of cAMP and was used as a positive control of PKA activation. After treatment, cell cultures were washed with PBS, scraped into a buffer containing 25 mM Tris-HCl, pH 7.4, 0.5 mM EDTA, 0.5 mM EGTA, 1 μg/ml leupeptin and 1 μg/ml aprotinine, and homogenized with a Pellet pestle motor homogenizer. This material was used for PKA activity determinations. Histone H1 (0.5 μg/μl) was incubated with 35 μg of cellular protein in a reaction mixture consisting of 50 mM Tris-HCl, 10 mM MgCl_2_, 100 μM ATP and 20 μCi of [^32^P]-ATP (200 μl of total volume). After 30 min of incubation, proteins were precipitated with 10% trichloroacetic acid, after adding bovine serum albumin (0.3 μg/μl), and then were centrifuged at 5,000 g for 10 min. Precipitated proteins were spotted onto p81 Whatman paper, washed in 75 mM orthophosphoric acid and dried before counting the radioactivity in a β counter. Results are expressed by rate of PKA activity with or without cAMP (1 μM).

### Clonogenic assay

Cytostatic effects of [V^4^Q^5^]dDAVP were also examined by the colony formation assay (22). MDA-MB-231 cells were grown in complete medium with dDAVP or [V^4^Q^5^]dDAVP (100–1,500 nM). Complete medium with tested peptides was renewed after 72 h. Seven days after cell seeding, colonies of >50 cells were counted. The concentration producing 50% inhibition (IC_50_) was determined by plotting a linear regression curve.

### Determination of cell cycle

Cell cycle was evaluated by flow cytometry (25). After 48 h of starvation, MDA-MB-231 cells were treated for 24 h with dDAVP or [V^4^Q^5^]dDAVP in complete medium and collected by trypsinization. Cells were then fixed in 70% chilled methanol for 30 min, treated with 1 μg/ml RNase A (Sigma-Aldrich) and stained with 100 μg/ml propidium iodide. Cell cycle phase distribution of nuclear DNA was carried out in a FACSCalibur cytometer using WinMDI 2.9 software.

### Endothelial cell morphogenesis assay

*In vitro* endothelial cell morphogenesis assay was performed using Matrigel-coated 24-well plates (BD Biosciences, San Jose, CA, USA) (15). Briefly, 1×10^5^ HMVEC-L cells were incubated with dDAVP or [V^4^Q^5^]dDAVP at a concentration of 100 nM. After allowing capillary tube formation for 24 h, randomly chosen fields were photographed at magnification ×100, and quantification was conducted. The number of capillary-like tubes formed in control cultures was taken as 100%.

### Mice

Specific pathogen-free 8-week-old female BALB/c and athymic BALB/c (nu/nu) mice were purchased from UNLP (Universidad Nacional de La Plata, Buenos Aires, Argentina), and kept 5–8 mice per cage in our animal house facility at the National University of Quilmes. Food and water was provided *ad libitum* and general health status of the animals was monitored daily. All protocols were approved by the National University of Quilmes institutional Animal Care Committee.

### In vivo angiogenesis assays

To evaluate effects on MDA-MB-231-induced angiogenesis, a modified Matrigel plug assay was conducted. A mixture containing 500 μl of Matrigel, heparin (50 U/ml) and 4.5×10^6^ tumour cells was injected subcutaneously into BALB/c athymic mice. Treatment consisted of three weekly intravenous doses of dDAVP or [V^4^Q^5^]dDAVP (0.3 μg/kg).

Animals were sacrificed 14 days after cell injection. Plugs were recovered and scanned at high resolution. The extent of vascularisation was assessed by the amount of haemoglobin detected in the implants using the Drabkin method (Sigma-Aldrich). The mean optical density of plugs from control group was taken as 1 (relative haemoglobin content).

An intradermal angiogenesis assay was performed to test [V^4^Q^5^]dDAVP effect on early F3II tumour-induced vascularisation (15). F3II cells (2×10^5^) per site were inoculated in the flank of BALB/c mice in a DMEM and trypan blue solution. After 5 days, animals were sacrificed and skins were photographed. The vascular network around the tumour cell implant was quantified using a millimeter grid. Daily intravenous doses of dDAVP or [V^4^Q^5^]dDAVP (0.3 μg/kg) were administered throughout the experiment.

### Tumour progression

To generate breast cancer xenografts, a 300-μl suspension containing 5×10^6^ MDA-MB-231 cells in DMEM and Matrigel (1:1 volume ratio) was injected subcutaneously in BALB/c athymic mice. Tumours were measured periodically with a caliper and tumour volume was calculated by the formula: 0.52 × width^2^ × length. Treatment started 14 days after MDA-MB-231 cell inoculation, when tumours reached volumes of ~50 mm^3^. DDAVP or [V^4^Q^5^]dDAVP (0.3 μg/kg) were administered intravenously thrice weekly. When the control group (saline vehicle) reached a mean tumour volume of 200 mm^3^ and exhibited signs of ulceration and necrosis, animals were photographed and tumours from different experimental groups were removed, fixed with formalin and routinely processed for haematoxylin and eosin (H&E) staining in order to examine tumour histology and vascularisation. The effect of [V^4^Q^5^]dDAVP on survival was also evaluated. Animals in saline vehicle or [V^4^Q^5^]dDAVP treated groups were euthanized by cervical dislocation when the humane tumour burden limits (>1,000 mm^3^) were reached (26). To generate tumours in immunocompetent hosts, 2×10^5^ F3II cells were injected subcutaneously in syngeneic BALB/c mice. Treatment started 7 days later, when tumours reached volumes of ~50 mm^3^. DDAVP or [V^4^Q^5^]dDAVP were administered as mentioned above. On day 50, F3II tumour-bearing animals were sacrificed and necropsied. To investigate the presence of spontaneous metastases, lungs were removed, fixed in Bouin’s solution and macroscopic lung nodules were counted under a dissecting microscope.

### Experimental lung metastases assay

To evaluate [V^4^Q^5^]dDAVP effect on blood-borne metastases, 2×10^5^ F3II cells in DMEM were injected into the tail vein of mice (9). On day 21, lungs were excised, weighted, fixed in Bouin’s solution, photographed and lung nodules were counted. DDAVP or [V^4^Q^5^]dDAVP were administered at 0.3 μg/kg in two intravenous doses, the first at time zero and the second 24 h after cell injection.

### Toxicology studies

Acute toxicology studies were conducted at the National University of Litoral (Argentina). All procedures were approved by the Institutional Ethics and Security Committee and are consistent with the Guide for the Care and Use of Laboratory Animals (NRC 2011). Groups of 5 Wistar female rats received single intravenous doses of 1, 10 or 100 μg/kg of [V^4^Q^5^]dDAVP or dDAVP. A full clinical evaluation, including heart and respiratory rates, nervous system, motor activity, biochemical and haematological studies, was conducted at 1, 3, 6, 12, 24 and 72 h after drug administration. Body weight, food and water intake were monitored daily.

### Statistical analysis

PRISM 6, Version 6.01 (GraphPad Software Inc., La Jolla, CA, USA) was used to conduct all statistical analyses (IC_50_ values, one-way and two-way ANOVA and Student’s t-test). Tukey’s multiple comparisons test was used after ANOVA analysis. In tumour progression protocols, growth rates represent the slopes of the linear regressions of the tumour volumes over time. In Kaplan-Meier plots, log-rank test and Cox regression analysis was applied to establish the association of treatment with survival. Differences were considered statistically significant at a level of P<0.05. Data are presented as mean ± SD or SEM.

## Results

### V2r expression in breast cancer and microvascular cells

Expression of V2r in MDA-MB-231 and F3II cells was first confirmed by immunofluorescence (Fig. 1C). MCF-7, a cell line known to display vasopressin membrane receptors (6), was used as a positive control of V2r expression. HMVEC-L cells were also positive for the V2r, as documented previously by reverse transcription-PCR (27).

### Cytostatic effects of [V^4^Q^5^]dDAVP on human breast cancer cells

**Figure 2:**
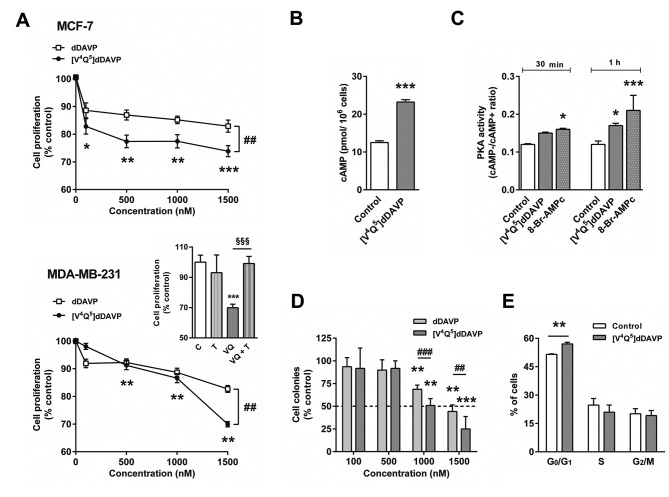
Effect of [V^4^Q^5^]dDAVP on *in vitro* growth of human breast cancer cells. (A) Antiproliferative effect of dDAVP or [V^4^Q^5^]dDAVP on log-phase growing MCF-7 (upper panel) and MDA-MB-231 (lower panel) human breast carcinoma cells. Inset for MDA-MB-231, blockade of antiproliferative effect of [V^4^Q^5^]dDAVP (1,500 nM) by the selective and competitive V2r antagonist tolvaptan (1,500 nM). C, control; T, tolvaptan; VQ, [V^4^Q^5^]dDAVP and VQ+T, [V^4^Q^5^]dDAVP plus tolvaptan. (B) cAMP concentration in MCF-7 cells after 1 h of [V^4^Q^5^]dDAVP treatment. (C) PKA activity in MCF-7 cells treated with [V^4^Q^5^]dDAVP (1,000 nM) or the membrane-permeable cAMP analogue 8-Br-cAMP (500 nM), an activator of PKA. (D) Effect of dDAVP or [V^4^Q^5^]dDAVP treatment on clonogenic growth of MDA-MB-231 cells. Dotted line indicates 50% inhibition. (E) DNA-cell cycle analysis of MDA-MB-231 cells treated with [V^4^Q^5^]dDAVP (1,000 nM) for 24 h. In all cases, data are presented as mean ± SEM. Results are representative of at least three independent experiments. ^*^P<0.05; ^**^P<0.01; ^***^P<0.001 versus control. ^##^P<0.01; ^###^P<0.001 dDAVP versus [V^4^Q^5^]dDAVP. ^§§§^P<0.001 [V^4^Q^5^]dDAVP plus tolvaptan versus [V^4^Q^5^]dDAVP alone.

We evaluated the cytostatic effect of the novel analogue [V^4^Q^5^]dDAVP and the parental peptide dDAVP on log-phase growing breast cancer cells (Fig. 2A). After a 72-h exposure, both peptides caused a mild reduction of proliferation in MCF-7 cell cultures (Fig. 2A, top). At concentrations >500 nM, [V^4^Q^5^]dDAVP treatment showed an enhanced cytostatic effect compared to dDAVP, reducing cell proliferation by ≤26%. These results are consistent with a previous study, where several dDAVP peptide analogues, including [V^4^Q^5^] dDAVP, were screened using MCF-7 cultures (21). Reduction of tumour cell proliferation by [V^4^Q^5^]dDAVP was associated with an activation of cAMP/PKA signalling axis. An increase in intracellular cAMP levels (Fig. 2B) and PKA activation (Fig. 2C) of nearly 90 and 40%, respectively, was observed after 60 min of incubation with [V^4^Q^5^]dDAVP. The cytostatic effect of the novel analogue was also evaluated in triple-negative MDA-MB-231 cells. The antiproliferative profile of [V^4^Q^5^]dDAVP was similar to the one obtained against MCF-7 cells (Fig. 2A, bottom). Growth-modulating activity was completely abolished by the selective V2r antagonist tolvaptan, indicating that reduction of cell proliferation mainly results from V2r activation (Fig. 2A, bottom inset). [V^4^Q^5^]dDAVP showed a much stronger effect on low density breast cancer cell cultures. MDA-MB-231 clonogenic growth was inhibited by 75% after 7-day treatment with 1,500 nM [V^4^Q^5^]dDAVP. Compared to parental peptide, novel analogue displayed an enhanced inhibitory effect on colony formation, with IC_50_ values of 1,130 and 1,440 nM for [V^4^Q^5^]dDAVP and dDAVP, respectively (Fig. 2D). Cell cycle distribution analysis showed that a 24-h treatment with [V^4^Q^5^]dDAVP (1,000 nM) resulted in partial arrest of MDA-MB-231 cells in G0/G1 phase (Fig. 2E).

### [V^4^Q^5^]dDAVP anticancer effects on human breast cancer xenografts

**Figure 3:**
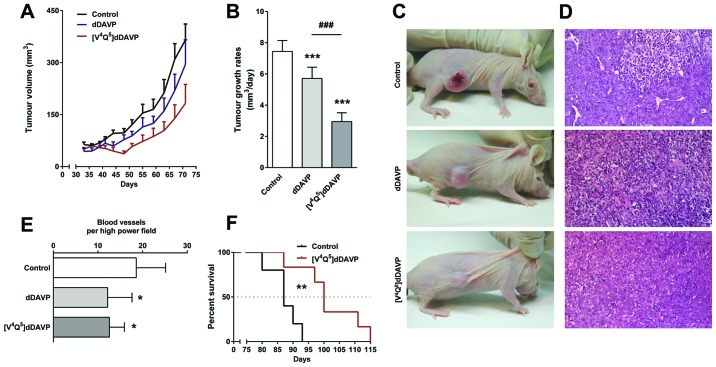
Effect of [V^4^Q^5^]dDAVP on tumour progression of human breast cancer xenografts. (A) MDA-MB-231 tumour volume in mice receiving saline vehicle (control), dDAVP or [V^4^Q^5^]dDAVP over time. (B) Growth rates of tumours from different experimental groups. (C) Representative photographs of nude mice bearing MDA-MB-231 xenografts. (D) Representative images of H&E stained tumour tissue sections. Magnification, ×200. (E) Quantification of microvessel density. (F) Kaplan-Meier survival plot for vehicle- or [V^4^Q^5^]dDAVP-treated groups. Dotted line indicates 50% survival. Data are presented as mean ± SEM (tumour volume curve) or SD (growth rates and blood vessel quantification). n=5 or 6 animals per experimental group. Tumour growth results are representative of two independent experiments. ^*^P<0.05; ^**^P<0.01; ^***^P<0.001 versus control and ^###^P<0.001 [V^4^Q^5^]dDAVP versus dDAVP.

We next evaluated the novel analogue on MDA-MB-231 xenograft growth. Treatment with thrice weekly intravenous doses of [V^4^Q^5^]dDAVP for 8 weeks reduced final tumour load by 50% (Fig. 3A). Tumours grew at rates of 2.95±0.56, 5.72±0.72 and 7.44±0.66 mm^3^/day (mean ± SD, P<0.001) in [V^4^Q^5^]dDAVP-treated, dDAVP-treated and control mice, respectively (Fig. 3B). In controls, xenografts grew by invading the subcutis and dermis, causing visible skin ulceration and necrosis. On the other hand, most animals treated with [V^4^Q^5^]dDAVP or dDAVP displayed preservation of superficial layers of skin, indicating inhibition of tumour infiltration and modulation of tumour aggressiveness (Fig. 3C). Histopathological studies of MDA-MB-231 xenografts from treated mice showed a decrease in tumour vascularisation (Fig. 3D). Quantification of intratumoural vascular density revealed a 30% reduction in the number of blood vessels in [V^4^Q^5^]dDAVP- and dDAVP-treated animals (Fig. 3E). As shown in the Kaplan-Meier curve, [V^4^Q^5^]dDAVP treatment was associated with increased survival (Fig. 3F).

### Reduction of angiogenesis by [V^4^Q^5^]dDAVP

**Figure 4:**
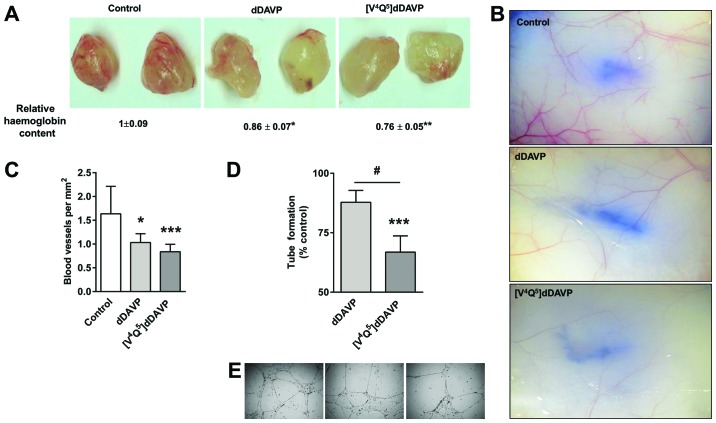
Effect of [V^4^Q^5^]dDAVP on *in vitro* and *in vivo* angiogenesis. (A) MDA-MB-231 cell-induced angiogenesis evaluated by a modified-Matrigel plug assay. Representative images of plugs recovered from vehicle, dDAVP or [V^4^Q^5^]dDAVP (0.3 μg/kg i.v.) treated mice are depicted. Relative haemoglobin content in Matrigel plugs was determined using the Drabkin’s method. (B) *In vivo* microvessel density in BALB/c immunocompetent mice intradermally inoculated with F3II mammary carcinoma cells and treated with daily dDAVP or [V^4^Q^5^]dDAVP (0.3 μg/kg i.v.) for 5 days. Representative images of F3II tumour cell-induced angiogenesis in different experimental groups. (C) Quantification of microvessel density around the F3II tumour cell implant. (D) Quantification of tube formation by HMVEC-L cells treated with dDAVP or [V^4^Q^5^]dDAVP. (E) Representative images of endothelial cell sprouting and tube formation. Magnification, ×100. For *in vivo* experiments, n=5–7 animals per experimental group. Data are presented as mean ± SEM (tube formation assay) or ± SD (intradermal angiogenesis assay and Matrigel plug assay). ^*^P<0.05; ^**^P<0.01; ^***^P<0.001 versus control. ^#^P<0.05 dDAVP versus [V^4^Q^5^]dDAVP.

To further evaluate the efficacy on angiogenic response, a modified Matrigel plug assay was used. Thrice weekly intravenous doses of [V^4^Q^5^]dDAVP or dDAVP resulted in a decrease in MDA-MB-231 cell-induced angiogenesis (Fig. 4A). In addition, the highly aggressive mammary carcinoma F3II cell line was intradermally injected and used to assess the effect on early tumour-induced vascular development. After 5 days, F3II cells generated highly irregular and dense vascular networks around tumour cell implants in control animals. In [V^4^Q^5^] dDAVP-treated mice, tumour angiogenesis was drastically inhibited, showing a vessel density reduction of ~50% (Fig. 4B and C). We also investigated direct effects of [V^4^Q^5^]dDAVP on microvascular endothelial cell morphogenesis (Fig. 4D and E). Twenty-four-hour incubation with [V^4^Q^5^]dDAVP reduced capillary-like tube formation by HMVEC-L cells. The parental peptide dDAVP did not displayed any significant effects on *in vitro* angiogenesis.

### Effects of [V^4^Q^5^]dDAVP on metastasic progression of F3II mouse mammary tumours

**Figure 5:**
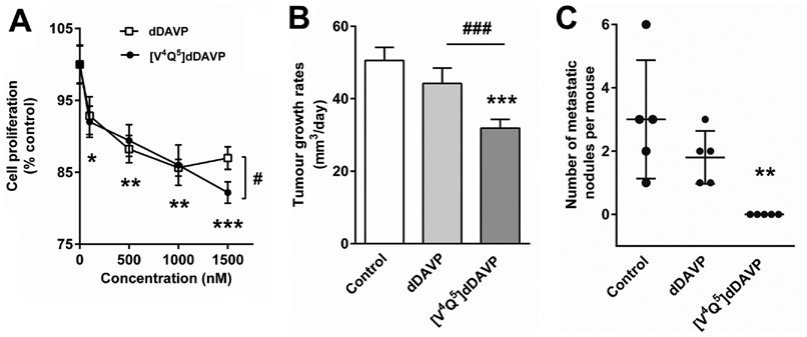
Effect of [V^4^Q^5^]dDAVP on tumour growth and spontaneous metastatic progression of subcutaneous mammary tumours. (A) Antiproliferative effect of dDAVP or [V^4^Q^5^]dDAVP on *in vitro* growth of V2r-expressing F3II cells. (B) After F3II tumours were generated in BALB/c immunocompetent mice, animals were treated with dDAVP or [V^4^Q^5^]dDAVP thrice a week (0.3 μg/kg i.v.). Tumour growth rates from day 11 onwards are shown. (C) Spontaneous lung metastases quantification. For *in vivo* experiments, n=5 animals per experimental group. Data are presented as mean ± SEM (cell proliferation assay) or ± SD (tumour growth rates and spontaneous metastases). ^*^P<0.05; ^**^P<0.01; ^***^P<0.001 versus control and ^###^P<0.001 [V^4^Q^5^]dDAVP versus dDAVP.

We first evaluated the *in vitro* response to [V^4^Q^5^]dDAVP and dDAVP in log-phase growing hormone-independent F3II cells. Treatment during 72 h with both peptidic compounds caused a mild cytostatic effect in a concentration-dependent manner (Fig. 5A). We further tested the effects on progression of F3II tumours in BALB/c mice. Both dDAVP and [V^4^Q^5^]dDAVP treatments had limited impact on final tumour volume (1194±240, 1574.8±466.1 or 1822.3±1185 mm^3^ in [V^4^Q^5^]dDAVP, dDAVP or control group, respectively, mean ± SD, P>0.05). Notwithstanding, tumours from [V^4^Q^5^]dDAVP-treated mice grew at a lower rate compared to tumours from animals administered with dDAVP or saline vehicle (Fig. 5B). All control animals displayed visible lung metastases, with a maximum of 6 macroscopic nodules per mouse. Remarkably, treatment with intravenous doses of [V^4^Q^5^]dDAVP for 6 weeks was able to completely abolish the formation of lung metastases (Fig. 5C). On the contrary, the effects of dDAVP on spontaneous metastases were not significant in the present experimental conditions.

### Inhibition of experimental lung metastases by [V^4^Q^5^]dDAVP

**Figure 6:**
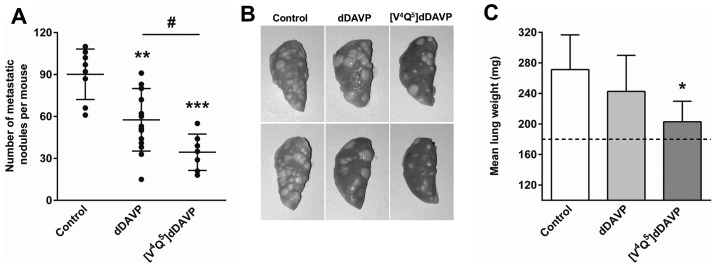
Effect of [V^4^Q^5^]dDAVP on experimental lung colonisation by F3II mouse mammary carcinoma cells. (A) Number of metastatic lung nodules per mouse. (B) Representative left lung lobes (two from each group) are depicted. (C) Average lung weights corresponding to the different experimental groups. The dotted line represents the average pulmonary weight of healthy BALB/c mice described by Han *et al* (28). n=8 animals per experimental group. Data are presented as mean ± SD ^*^P<0.05; ^**^P<0.01; ^***^P<0.001 versus control. ^#^P<0.05 [V^4^Q^5^]dDAVP versus dDAVP.

F3II cells inoculated intravenously into BALB/c mice induce multiple macroscopic lung lesions after 3 weeks. Experimental lung colonisation by F3II cells was severely impaired after either dDAVP or [V^4^Q^5^]dDAVP treatment. However, whilst dDAVP reduced the number of lung nodules by 32%, the novel analogue [V^4^Q^5^]dDAVP displayed greater antimetastatic efficacy, reducing by 62% the formation of metastases (Fig. 6A and B). In addition, total lung weight was significantly reduced in [V^4^Q^5^]dDAVP-treated animals (Fig. 6C), with a weight close to healthy lung in BALB/c mice (28).

### Toxicology studies

Preliminary acute toxicology studies conducted in Wistar rats revealed that intravenous administration of [V^4^Q^5^]dDAVP at doses of 1, 10 and 100 μg/kg had no influence on general symptoms, body weight, food and water consumption (Table I). These observations suggest that individual injections are safe at doses ≥300-fold above that required for antiangiogenic/antimetastatic effects. Mild transient increases of glycemia and bilirubin were observed in treated groups. The other biochemical and haematological parameters were not significantly altered. DDAVP was administered as a reference standard, showing a safety profile consistent with previous observations (13,15).

## Discussion

Vasopressin and its receptors were proposed as attractive targets for breast cancer therapy almost two decades ago, when vasopressin gene-related products were detected by immunohistochemistry as a feature of all breast cancer subtypes (29). Selective agonists of V2 vasopressin membrane receptor, such as dDAVP, seem to evoke dual angiostatic and antimetastatic effects, breaking co-operative interactions of tumour and endothelial cells during tumour progression (18). Due to the interesting anticancer activity of dDAVP in animal studies (9,11,12,15), as well as its known haemostatic properties (3), a prospective, open-label phase II clinical trial is currently ongoing with the aim of assessing safety and preliminary anticancer efficacy of perioperative use of dDAVP in breast cancer patients (NCT01606072). Peptides such as dDAVP are much appreciated as lead compounds for the development of new drugs with enhanced biological activity. In the present study, we characterized and compared the preclinical anticancer efficacy of the novel analogue [V^4^Q^5^]dDAVP with its parental peptide dDAVP. [V^4^Q^5^]dDAVP was originally selected from a panel of peptidic analogues derivatized from dDAVP with different sequence and structural modifications, mainly aiming at the N-terminal loop of the molecule. This search for more potent and selective V2r agonists included full-length nonapeptides, tetrapeptides and chiral isomers (21).

In the present study, *in vitro* studies exhibited moderate cytostatic activity of [V^4^Q^5^]dDAVP on log-phase growing human breast cancer cells, but showed a more potent inhibitory effect on low density cell culture, with an IC_50_ value of 1.13 μM. Chemical V2r blockade by tolvaptan completely abolished [V^4^Q^5^]dDAVP effects in MDA-MB-231 cells. These findings are in close agreement with the study by Keegan *et al (*30), where mild cytostatic effects of dDAVP on breast cancer cells were blocked by satavaptan, another non-peptidic V2r antagonist. Action of [V^4^Q^5^]dDAVP was associated with partial cell cycle arrest and normal V2r-activated signal transduction, involving intracellular cAMP elevation and PKA activation. Increases in cAMP intracellular levels using cAMP analogues or cAMP elevating agents, such as hormones or forskolin, can trigger cell cycle arrest or proapoptotic responses in numerous cancer cell types, including breast cancer (31–33). Consequently, several authors have postulated the adenylate cyclase/cAMP/PKA axis as a growth suppressor system in breast cancer that could be targeted to block tumour formation (33,34).

Triple-negative breast cancer is defined by a lack of expression of both PR and ER, as well as a low expression of (HER2)/neu (35). There have been significant improvements in the outcome of other subtypes of breast cancer, including ER-positive/HER2 overexpressed tumours, attributed to the addition of targeted therapy, including hormonal agents and trastuzumab/pertuzumab (36,37). However, no targeted therapies are available for the treatment of triple-negative breast cancer, and frontline treatments are limited to surgical approaches and chemotherapeutics (38). In the present study we documented the efficacy of [V^4^Q^5^]dDAVP on triple-negative MDA-MB-231 breast cancer xenografts. Intravenous injection of clinically-relevant doses of [V^4^Q^5^]dDAVP caused a marked reduction in tumour growth and an increase in survival. Histological examination of xenografts also showed a significant decrease in tumour angiogenesis in treated animals. In a previous study, our group reported that i.v. administration of dDAVP at doses of 2 μg/kg was able to significantly reduce intratumour vascularisation of F3II mammary tumours (15). DDAVP seems to modulate tumour angiogenesis by inducing the formation of angiostatin, a potent angiogenesis inhibitor that is generated by cancer-mediated proteolysis of plasminogen (16,17). Interestingly, an enhanced production of angiostatin by human mammary carcinoma cells was previously reported after incubation with [V^4^Q^5^]dDAVP compared to dDAVP-treated cells (39). Nevertheless, the possibility that other underlying mechanisms account for the antiangiogenic action of dDAVP or [V^4^Q^5^]dDAVP cannot be ruled out. Systemic injection of dDAVP induces a rapid release of VWF by stimulation of V2r present in microvasculature. VWF is a large multimeric plasma glycoprotein that plays an essential role in primary haemostasis. This factor acts as a carrier for coagulation factor VIII and mediates platelet adhesion to endothelial cells (27,40). Starke *et al* reported that loss of endothelial VWF by short interfering RNA results in increased *in vitro* angiogenesis. Additionally, VWF-deficient mice displayed increased mature blood vessel density, suggesting a potential role for VWF in the modulation of angiogenesis (41). Other possible mechanism involves V2r-related signalling and actin. Stimulation of V2r in endothelial cells leads to activation of cAMP-mediated signalling, which plays a central role in actin cytoskeletal dynamics and cell migration (27,42,43). Interestingly, it has been reported that PKA activation suppresses endothelial cell migration *in vitro* and angiogenesis *in vivo* (44). The connection between the cAMP signalling pathway and actin structures could partially explain the direct effects of [V^4^Q^5^]dDAVP on HMVEC-L tube formation. However, the specific mechanisms responsible for [V^4^Q^5^]dDAVP effects on microvascular endothelial cells remain to be elucidated.

The F3II breast cancer cell line is invasive and metastatic, characterized by an aggressive hormone-independent growth and a low expression of (HER2)/neu, as revealed by immunocytochemistry studies. In addition to angiostatic effects, [V^4^Q^5^]dDAVP treatment of immunocompetent mice bearing F3II tumours resulted in complete inhibition of metastatic progression. [V^4^Q^5^]dDAVP also showed an enhanced antimetastatic effect compared to dDAVP on experimental metastases to lung. As mentioned above, dDAVP causes the release of multimeric forms of VWF, reaching peak levels at 60–90 min after i.v. injection (4). Using VWF-deficient mice, Terraube *et al* demonstrated that the absence of VWF leads to increased metastatic potential of intravenously injected carcinoma cells. Furthermore, VWF was shown to directly induce apoptosis of tumour cells *in vitro* and caused death of metastatic cells arrested in the lungs (14,45). By modulating the interaction between cancer cells and subendothelial cells, VWF seems to reduce sustained adherence of tumour cells in the microvasculature at the target organ, thus inhibiting metastatic spread. More recently, it was found that aggressive breast and lung cancer cells with high levels of ADAM28 (a disintegrin and metalloproteinase 28) are able to avoid VWF-induced apoptosis at micrometastatic sites. ADAM28 binds and cleaves VWF, thus favoring the survival of metastatic cells in the tissue microenvironment (46). Taken together, these results suggest that VWF released after V2r stimulation plays a crucial role in resistance to blood-borne metastases.

Hydrophobicity enhancement at position 4 (glutamine by valine) and conservative substitution at position 5 (asparagine by glutamine) in [V^4^Q^5^]dDAVP resulted in an improved antitumour compound derived from dDAVP. The V2r is a transmembrane receptor that belongs to the G protein-coupled receptor family, having a deep cavity on the extracellular side containing hydrophobic moieties (19,20). Manning *et al* hypothesized that enhancing hydrophobicity at position 4 improves the interaction of vasopressin-related ligands with V2r (2). In a separate study, Manning and collaborators reported that 4-valine-dDAVP has a 10-fold higher affinity for the human V2r than dDAVP, with Ki values of 2.2 and 23.3 nM, respectevely (5). More recently, it was shown that, unlike dDAVP, 4-valine-dDAVP administration was able to rescue the function of a mutated V2r in a pathological setting, displaying an enhanced agonistic potency on intracellular cAMP production without cross-reacting with other vasopressin receptor subtypes (47). In order to improve the stability of the analogue, we also introduced a conservative substitution at position 5, replacing asparagine with glutamine, based on its distinctive susceptibility to the deamidation process. Although both asparagine and glutamine are susceptible to deamidation, deamidation of glutamine proceeds at a much slower rate than deamidation of asparagine at peptide level (48,49).

These rational modifications may favour ligand-receptor affinity by promoting hydrophobic interactions between the N-terminal conformational loop of [V^4^Q^5^]dDAVP and the amino acids located at the bottom of the V2r cavity. Enhancement of affinity between [V^4^Q^5^]dDAVP and V2r present in cancer and endothelial cells could result in augmented cAMP production and PKA activation, angiostatin generation and VWF release, thus explaining the increased biological activity of the peptidic analogue. Nevertheless, further pharmacological experiments should be performed to confirm stability, selectivity and potency of the novel compound.

In conclusion, compared to dDAVP, the novel analogue [V^4^Q^5^]dDAVP exhibited a significantly higher inhibitory effect on breast cancer cell proliferation and colony formation, as well as on MDA-MB-231 and F3II tumour growth. [V^4^Q^5^]dDAVP also displayed greater antimetastatic effects than dDAVP on spontaneous and experimental lung colonization.

While treatment for localised tumours has generally improved survival in the era of modern medicine, patients with advanced stage metastatic disease still suffer from a lack of effective therapies. Despite evident progress in overall mortality, the efficacy of adjuvant chemotherapy in reducing metastatic risk has reached a plateau (50). Given that the benefit of chemotherapy is often mitigated by long-term side effects linked to a lack of selectivity (51–53), the potential combination with novel highly selective cytostatic agents such as [V^4^Q^5^]dDAVP is highly interesting. Preclinical efficacy of the compound without overt toxicity supports further clinical development of [V^4^Q^5^]dDAVP as a novel adjuvant or maintenance therapy in aggressive and metastatic hormone-independent breast cancer.

## Figures and Tables

**Table I:** Acute toxicology study in Wistar rats after single intravenous doses of 1, 10 or 100 μg/kg of dDAVP or [V^4^Q^5^]dDAVP.

	Experimental groups						
	
Parameter^a^	Control	dDAVP 1 μg/kg	dDAVP 10 μg/kg	dDAVP 100 μg/kg	[V^4^Q^5^]dDAVP 1 μg/kg	[V^4^Q^5^]dDAVP 10 μg/kg	[V^4^Q^5^]dDAVP 100 μg/kg
Weight^b^ (g)	218.3±8.7	234.2±8.1	240.3±9.4	241.8±16.8	225.4±16.8	226.0±10.9	225.8±6.1
Hematocrit^c^ (%)	39.3±4.0	40.4±2.3	40.5±2.7	41.8±3.4	40.0±1.6	41.0±2.2	41.2±1.7
RBC (10^6^/ml)	5.1±0.5	5.2±0.3	5.5±0.3	5.7±0.3	5.6±0.2	5.4±0.5	5.5±0.2
WBC (10^3^/ml)	4.8±0.4	4.8±0.3	4.4±0.5	4.1±0.7	4.2±0.2	4.5±0.3	4.7±0.7
Fibrinogen^c^ (mg/dl)	198.3±44.8	221.0±67.8	226.7±48.4	243.0±63.1	206.0±56.7	210.0±41.8	179.5±45.7
Total protein^c^ (g/dl)	7.3±0.6	6.6±0.8	6.9±0.5	7.0±0.5	6.9±0.5	6.7±0.6	6.9±0.5
Direct bilirubin (mg/dl)	0.08±0.02	0.13±0.04	0.14±0.02^d^	0.17±0.03^d^	0.11±0.01^d^	0.12±0.02^d^	0.16±0.05^d^
Glucose (mg/dl)	179.9±4.1	207.0±25.7^d^	218.8±20.0^d^	230.7±12.1^d^	217.7±13.9^d^	221.1±19.7^d^	230.2±12.1^d^
Creatinine (mg/ml)	0.69±0.27	0.70±0.16	0.65±0.08	0.70±0.09	0.58±0.03	0.64±0.07	0.61±0.05
GGT (IU/l)	2.7±0.6	2.8±0.8	2.6±0.9	3.0±1.2	2.8±0.8	3.2±1.1	3.6±0.9
AST (IU/l)	34.7±6.4	36.6±11.6	30.0±5.6	47.5±17.8	39.2±6.7	51.2±30.6	46.6±20.1
ALT (IU/l)	19.0±1.0	22.8±3.4	22.6±3.4	21.3±2.1	20.6±2.7	19.6±3.1	20.4±3.1

a Toxicology parameters were measured 72 h after intravenous administration of the drugs unless stated otherwise.

b Body weight was also monitored 24 and 48 h after drug injection.

c For haematocrit, fibrinogen and total protein measurements blood samples were also collected and analysed at 1, 3, 6, 10 and 24 h after intravenous administration of the drugs. No significant changes were observed between groups (data not shown). RBC, red blood cells; WBC, white blood cells; GGT, γ-glutamyl transpeptidase; AST, aspartate aminotransferase; ALT, alanine aminotransferase. Values represent mean ± SD; and

d P<0.05 (ANOVA). Number of rats per experimental group, 5–6.
